# “Click on the bidirectional switch”: the aptasensor for simultaneous detection of lysozyme and ATP with high sensitivity and high selectivity

**DOI:** 10.1038/srep18814

**Published:** 2016-01-08

**Authors:** Feng Chen, Changqun Cai, Xiaoming Chen, Chunyan Chen

**Affiliations:** 1 Key Laboratory of Environmentally Friendly Chemistry and Applications of Ministry of Education, College of Chemistry, Xiangtan University, Xiangtan, Hunan 411105, China

## Abstract

A bifunctional and simple aptasensor was designed to one-spot simultaneously detect two analytes, lysozyme and ATP. The aptasensor was obtained by the electronic interaction between methyl violet (MV) and dsDNA. The dsDNA was obtained by hybridization of ATP aptamer and lysozyme aptamer. And we used the resonance light scattering (RLS) technique to detect the concentration of lysozyme and ATP. During the procedure of detection, the aptasensor works like a bidirectional switch, the corresponding side of the dsDNA will open when the target (lysozyme or ATP) “click” the aptamer, which results in corresponding RLS signal change. By the combination of the RLS technique, it is found that the changed RLS intensity was proportional to the concentration of lysozyme and ATP. The mixtures of ATP and lysozyme also met two binary function relations. The results indicated that the aptasensor could achieve simultaneous detection of ATP and lysozyme, the detection limits of ATP and lysozyme could reach 10^−11^ M and 10^−12^ M, respectively. The aptasensor shows potential application for small molecule and protein detection by RLS, it could extend the application of RLS technique.

Aptamers have been shown to bind their targets with high specificity and affinity, many aptamers become more widely used for any given molecular target, from metal ions and small chemicals to large proteins and higher order protein complexes, whole cells or viruses. However, only a few aptasensors, such as optically or electrochemically encoded quantum dots^1^,^2^,^3^,^4^, upconversion nanoparticles^5^,^6^, dyes^7–10^ for parallel analysis of different analytes have been developed. However, the shortcomings of these aptasensors, such as the functionalized aptamers, the interference between signal-transduction labels, relatively high cost, hard manipulation, time-consuming, have limited their wide applications. Because of the advantages of rapidity and high sensitivity, RLS technique has more widely applications including the detection of DNA^11^,^12^, proteins^13^,^14^, small biological molecules^15^, and metal ions^16^. All these aptasensors for parallel analysis of different analytes have not used RLS technique. In this work, we reported a simple aptasensor (Fig. 1) for one-spot simultaneous detection of lysozyme and ATP by using RLS technique. The aptasensor was obtained by the electronic interaction between MV and dsDNA. The dsDNA was obtained by hybridization of ATP aptamer and lysozyme aptamer. The obtained aptasensor was label-free and intellgent. It works like a bidirectional switch: when ATP is added, one side of the dsDNA is opened by the specifical binding of ATP-aptamer with ATP, resulted in RLS declined; when lysozyme is added, the other side of dsDNA is opened by the specifical binding of lysozyme-aptamer with lysozyme, resulted in RLS enhanced. Especially, for the first time, this aptasensor can be used to one-spot simultaneously detect the two analytes with excellent selectivity and high sensitivity.

Two analytes, lysozyme and ATP, were simultaneously detected in this aptasensor. Lysozyme can aggregate a variety of micriirganisms, which can promote the clearance of bacteria from the oral cavity^17^,^18^,^19^. The abnormal concentration of lysozyme in serum and urine is related to many diseases, such as leukemia, renal diseases, and meningitis, and it has been used as an important biomarker^20^. Hence, several methods have been used for the detection of lysozyme, including resonance Rayleigh scattering^21^, electrochemistry^22^, surface plasmon resonance^23^, fluorescence^24^ and molecularly imprinting technology^25^. ATP is the primary energy resources for numerous reactions in organisms including microtubule assembly, insulin secretion, ion channel regulation, and so on. ATP plays critical role in the organisms, for which many assays have been developed to monitor its concentration: the electrochemical assay^26^, the fluorescence assay^27^, the chemiluminescence assay^28^.

Sensitive and selective simultaneous detection of ATP and lysozyme are very important in biomedical research and clinical diagnosis. Aptamer-based sensors have been made on multiplexed detection of ATP and lysozyme and their analogues, including electrochemistry^4^,^10^,^29^,^30^,^31^,^32^,^33^,^34^ fluorescence^35^,^36^,^37^ and surface-enhanced Raman scattering (SERS)^38^. For example, Xiang *et al.* developed an electrochemical aptamer-based sensing platform for one-spot simultaneous determination of lysozyme and adenosine^31^. In their report, aptamers were labeled with methylene blue and ferrocene. However, such labeling has disadvantages that the labeling process makes the experiments relatively complex and expensive and also affects the binding affinity between the targets and their aptamers to a certain degree. Chen *et al.* developed a fluorescent probe for the detection ATP and thrombin by monitoring the fluorescent intensities with the corresponding Cy5 and TAMRA^35^. In their report, six DNA strands were designed and hybridized to form a closed state, and the aptamers were respectively immobilized on magnetic beads, however, multiple DNA strands makes the experiments complex and expensive. Zhang *et al.* designed a SERS bio-barcodes probe for the detection of the ATP and lysozyme with high sensitivity, but needed multiple amplification steps and one magnetic-separation procedure^38^. Compared with these multiplexed detection mothods, our new RLS-based aptasensor exhibited several advantages. Firstly, the sensor could one-spot simultaneously detect ATP and lysozyme with high accuracy. Secondly, the procedure was significantly simplified without the need for cumbersome substrate materials preparation. Finally, the sensor exhibited not only high sensitivity and specificity, with the detection limits of ATP and lysozyme for 10^−11^ M and 10^−12^ M, respectively, but also has good reproducibility. Besides, the sensor was very simple, available and low-cost. Because of these advantages, this sensor provided a promising potential application for small molecule and protein detection, and also extended the application of RLS.

## Results and Discussion

### Design and Preparation of the Aptasensor

#### Explanation of Construction of the Aptasensor

##### The enhanced RLS intensity by MV

**Figure 1:**
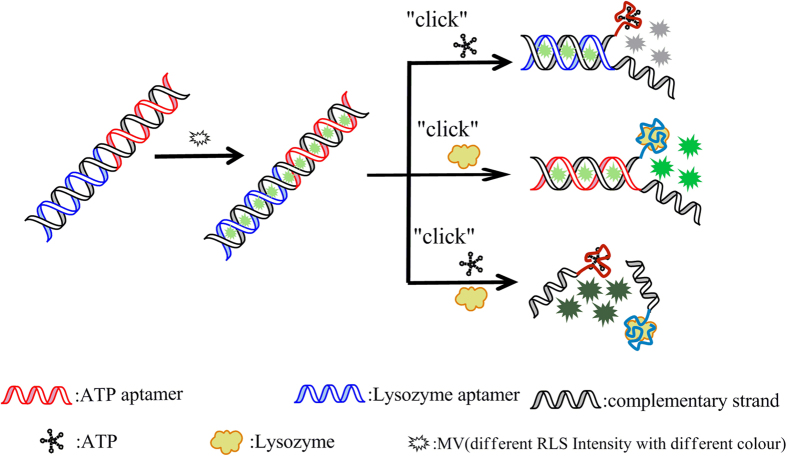
Schematic Representation of the Overall RLS-Based Detection Scheme of ATP and Lysozyme.

**Figure 2:**
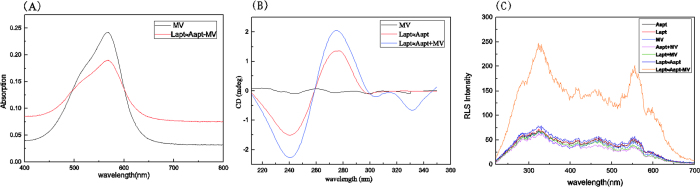
(**A**) Absorption spectra of bindings of MV with Lapt≈Aapt. Conditions: C_MV_:2.0 × 10^−6^ M; C_Aapt_ = C_Lapt_:1.0 × 10^−8^ M, pH = 7.4 (**B**) CD spectra of bindings of Lapt  ≈Aapt with MV. Conditions: C_MV_:4.0 × 10^−4^ M; C_Aapt_ = C_Lapt_:2.0 × 10^−6^ M, pH = 7.4 (**C**) RLS spectra of bindings of Lapt≈Aapt with MV. Conditions: C_MV_:2.0 × 10^−6^ M; C_Aapt_ = C_Lapt_:1.0 × 10^−8^ M, pH = 7.4

Figure 1 MV is one kind of triphenyl methane dye (TMR). TMR was embedded between a base quadruple and base pair and suggested that ligand binding was stabilized by base-stacking interactions^39^. To verify the bindings of MV with dsDNA, absorption and circular dichroic (CD) spectrum of the detection system was tested. The absorption spectra (shown in Fig. 2(A)) showed that no new peaks appeared and the maximum absorbance wavelength of MV did not change, but the absorbency of MV remarkably declined in the presence of dsDNA. The hypochromicity suggested that MV would interact with dsDNA due to the electronic interaction of the MV within the DNA bases^40^. In Fig. 2(B), a band with negative and positive peaks around 245 and 275 nm have been observed, which is typical of CD spectrum of DNA^41^. And a weaker, negative induced CD signal around 330 nm is also observed for the interaction of MV with dsDNA, which would be expected for an intercalative-binding mode^42^,^43^. Furthermore, the observation of the increased CD signal around 280 nm is important evidence for the interaction of MV with the base pairs^44^. From the RLS spectra (shown in Fig. 2(C)), it could be seen that the RLS signals of MV, lysozyme aptamer (Lapt), ATP aptamer (Aapt), MV+Lapt, MV+Aapt and Lapt≈Aapt (Lapt≈Aapt, a kind of dsDNA, formed by Lapt and Aapt, herein we used “≈” to indicate a double strand) were weak over the range of 200.0–700.0 nm, while the RLS signals of Lapt≈Aapt-MV was strong over the range of 200.0–700.0 nm. The result showed that the interaction between MV and dsDNA resulted in amplified resonance light scattering signals.

##### The changed RLS signal by the aptamer-target complex

**Figure 3:**
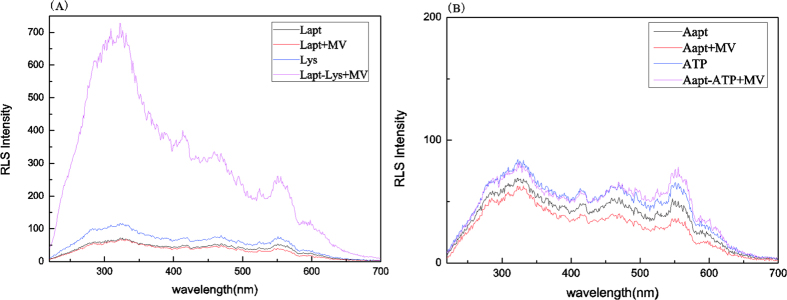
(**A**) RLS spectra of binding of Lapt with lysozyme. (**B**) RLS spectra of binding of Aapt with ATP. Conditions: C_MV_:2.0 × 10^−6^ M; C_Aapt_ = C_Lapt_:1.0 × 10^−8^ M; C_ATP_:5 × 10^−7^ M; C_lysozyme_: 5 × 10^−7^ M, pH = 7.4.

Lapt folded into tertiary structures with lysozyme to form a larger volume complex Lapt-lysozyme by a number of driving forces, including hydrogen bonding, electrostatic interactions, van der Waals forces, and stacking interactions. According to the RLS theory, the strong RLS signal would be obtained if the molecular form aggregates. Therefore, the RLS spectra of binding of Lapt with lysozyme showed that greatly enhanced RLS signals could be observed by the complex Lapt-lysozyme (shown in Fig. 3(A)). While the RLS spectra of binding of Aapt with ATP showed that negligible change of RLS signals (shown in Fig. 3(B)).

##### Principle

The above result showed that the interaction between MV and dsDNA resulted in amplified resonance light scattering signals. In the presence of ATP, one side of dsDNA was opened by the specific binding of Aapt with ATP, the structure Lapt≈Aapt-MV would be broken, and resulted in RLS declined, and the new formed complex Aapt-ATP would not contribute to the RLS signal. Therefore, the declined RLS intensity was proportional to the concentration of ATP. In the presence of lysozyme, the other side of dsDNA was opened by the specific binding of Lapt with lysozyme, the structure Lapt≈Aapt-MV would be broken, and resulted in RLS declined, and the new formed complex Lapt-lysozyme had high RLS intensity. Due to the enhanced RLS signal was much bigger than the declined RLS signal, the enhanced RLS intensity was proportional to the concentration of lysozyme. In the presence of ATP and lysozyme, dsDNA would be opened by the specific binding of aptamers with targets, the formed complex Lapt-lysozyme resulted in RLS enhanced, the structure Lapt≈Aapt-MV resulted in RLS declined, so, the RLS signal was influenced by both lysozyme and ATP. This demonstrated the capability of the designed sensor for one-spot simultaneous RLS detection of lysozyme and ATP.

### Optimization of the assay conditions

In order to obtain a sensitive, simple and practical assay for ATP and lysozyme, the RLS detection conditions were optimized in this study. The effects of aptamer concentration, MV concentration, temperature, time and pH were shown in the supplementary material.

### RLS detection of ATP or Lysozyme

**Figure 4:**
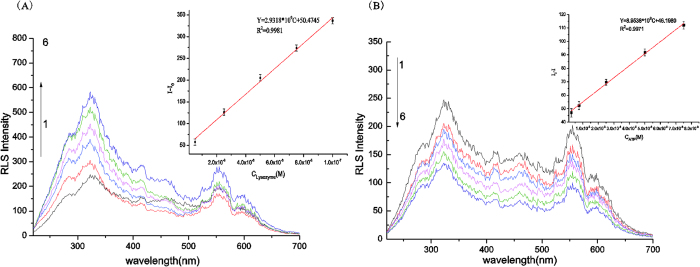
(**A**) RLS spectra and variance of normalized RLS intensity with the concentration of lysozyme. Conditions: C_lysozyme_:(1) 0 M (2) 5 × 10^−9^ M (3) 2.5 × 10^−8^ M (4) 5 × 10^−8^ M (5) 7.5 × 10^−8^ M (6) 1 × 10^−7^ M. (**B**) RLS spectra and variance of normalized RLS intensity with the concentration of ATP. Conditions:C_ATP_: (1) 0 M (2) 2.5 × 10^−9^ M (3) 7.5 × 10^−9^ M (4) 2.5 × 10^−8^ M (5) 5 × 10^−8^ M (6) 7.5 × 10^−8^ M, pH = 7.4.

Under the optimized conditions, the performance of the assay was evaluated by detecting the relationship between the changed RLS intensity (ΔI_RLS_) and the concentration of ATP or lysozyme standard solution. Figure 4(A) showed that the intensity of the RLS signals increased with the increase of the lysozyme concentration. Figure 4(B) showed that the intensity of the RLS signals declined with the increase of the ATP concentration. The strongest RLS band at 323 nm was used for the quantitative evaluation of ATP and lysozyme. For the detection of lysozyme, a series of lysozyme solutions from 5 × 10^−9^ to 1 × 10^−7^M were investigated and the results were shown in Fig. 4(A) The regression equation was ΔI = 2.9318 × 10^9^C + 50.4745 (C was the concentration of lysozyme, ΔI = I–I_0_, I was the RLS intensity after lysozyme, I_0_ was the RLS intensity before lysozyme, R = 0.9990), which showed a good linear relationship between peak height of RLS signal intensity and lysozyme concentration in the range from 5 × 10^−9^ to 1 × 10^−7^M, with a detection (LOD,3σ) of 9.3172 × 10^−12^ M. The relative standard deviation (RSD) was 0.91% obtained from 11 replicate measurements of 1 × 10^−7^M lysozyme, indicated a good reproducibility of the assay. For the detection of ATP, a series of ATP solutions from 2.5 × 10^−9^ to 7.5 × 10^−8^M were investigated and the results were shown in Fig. 4(B) The regression equation was ΔI = 8.9538 × 10^8^C + 46.1980 (C was the concentration of ATP, ΔI = I_0_−I, I_0_ was the RLS intensity before ATP, I was the RLS intensity after ATP, R = 0.9985), which showed a good linear relationship between peak height of RLS signal intensity and ATP concentration in the range from 2.5 × 10^−9^ to 7.5 × 10^−8^M, with a detection (LOD,3σ) of 4.6546 × 10^−11^ M. The relative standard deviation (RSD) was 1.39% obtained from 11 replicate measurements of 2.5 × 10^−8^M ATP.

### RLS detection of mixtures of ATP and Lysozyme

**Figure 5:**
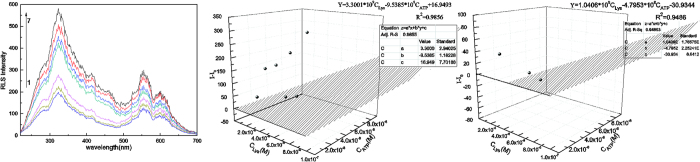
RLS spectra and the changed RLS intensity with the concentration of mixtures of ATP and Lysozyme. Conditions: (1) C_ATP_:8 × 10^−8^ M C_lysozyme_:2 × 10^−9^ M (2) C_ATP_:6 × 10^−8^ M C_lysozyme_:6 × 10^−9^ M (3) C_ATP_:2 × 10^−8^ M C_lysozyme_:1 × 10^−8^ M (4) C_ATP_:8 × 10^−9^ M C_lysozyme_:4 × 10^−8^ M (5) C_ATP_:4 × 10^−9^ M C_lysozyme_:6 × 10^−8^ M (6) C_ATP_:2 × 10^−9^ M C_lysozyme_:8 × 10^−8^ M (7) C_ATP_:1 × 10^−9^ M C_lysozyme_:1 × 10^−7^ M, pH = 7.4.

The above results showed that the RLS intensity change was proportional to the concentration of lysozyme or ATP. In this way, two-variable linear equation Z = ax + by + C was design for binary linear fit to the concentration of lysozyme, ATP and ΔI_RLS_ .With the concentration of lysozyme in the range from 2 × 10^−9^ to 1 × 10^−7^M and ATP in the range from 1 × 10^−9^ to 8 × 10^−8^ M, the changed RLS intensity at 323 nm was conformed to the relation (shown in Fig. 5): Δ*I*_RLS_ = 3.3001 × 10^9^C_Lysozyme_ − 9.5385 × 10^8^C_ATP_ + 16.9493 (R^2^ = 0.9856), the changed RLS intensity at 555 nm was conformed to the relation: Δ*I*_RLS_ = 1.0406 × 10^9^C_lysozyme_ − 4.7953 × 10^8^C_ATP_ − 30.9344 (R^2^ = 0.9486). The unique solution of the system of linear equations corresponded to the concentration of lysozyme and ATP, respectively. In this paper, a test was also defined to check the system of linear equations, making use of cross parameters methods.

### Selectivity of the assay

**Figure 6:**
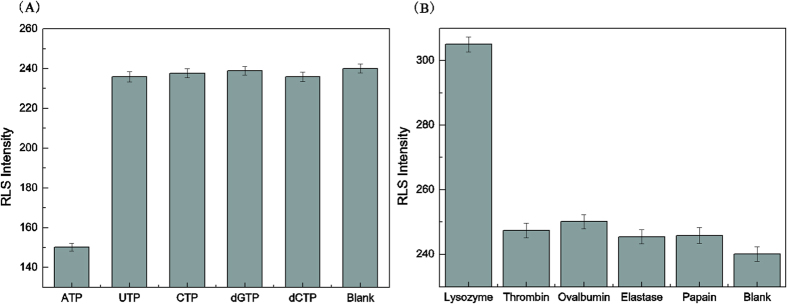
(**A**) Specificity for the detection of ATP. Conditions: C_ATP_:5 × 10^−8^ M C_UTP_ = C_CTP_ = C_dCTP_ = C_dGTP_:5 × 10^−6^ M. (**B**) Specificity for the detection of lysozyme. Conditions: C_lysozyme_:5 × 10^−9^ M C_Thrombin_ = C_Ovalbumin_  = C_Elastase_  = C_Papain_:5 × 10^−7^ M, pH = 7.4.

The specificity of the RLS-based aptasensor has been investigated and the corresponding results were shown in Fig. 6. UTP, CTP, dGTP and dCTP, which belong to the nucleoside family, were employed to assess the specificity of the aptasensor for the detection of ATP. Thrombin, ovalbumin, elastase and papain were chosen as controls to assess the specificity of the aptasensor for the detection of lysozyme. As show in Fig. 6, despite the existence of a large excess (100-fold) of the control molecules, UTP, CTP, dGTP and dCTP for ATP, or Thrombin, ovalbumin, elastase and papain against lysozyme, no significant increase of RLS signal was observed compared with the blank tests. However, the signal for lysozyme was much larger and the signal for ATP was much smaller and apparently distinguishable from the RLS intensity of the above. The experimental results demonstrated that the developed strategy exhibited a sufficient specificity for ATP or lysozyme detection, which was attributed to the specific binding between the target and its aptamer.

### Detection of ATP and lysozyme in serum sample

To investigate the feasibility of the proposed method for clinical chemistry, the performance of the assay in actual serum was tested by standard addition method. The human plasma was obtained from the Hospital of Xiangtan University and centrifuged at 5000 rpm for 8 min. Then, various concentrations of ATP and lysozyme were added in supernatant, and detected by RLS technique. The experimental results were shown in Table 1, indicating that the developed assay represents good recovery and offers great potential for specific detecting of lysozyme and ATP in biological fluids.

### Simultaneous detection of ATP and lysozyme in bacterial lysates

As the experimental data show, our proposed method works well for ATP and lysozyme assay in human serum systems. On the other hand, in this method, aptamer (DNA molecules) is used as the binding element of lysozyme and ATP. So, the effects of DNA endonucleases or exonuclease on the aptasensor should be investigated. Therefore, simultaneous detection of ATP and lysozyme was performed in bacterial lysate, which including these DNA enzymes. Table 2 shows that the concentration of lysozyme and ATP can be simultaneously quantitated in bacterial lysate. This observation suggests that the proposed method can simultaneously detect ATP and lysozyme in bacterial lysates without the interferences of these DNA enzymes. See the Supporting Information for the detailed descriptions of the bacterial culture and lysis.

## Materials and Methods

### Materials

The oligonucleotides with the following sequences: 5′-ATC TAC GAA TTC ATC AGG GCT AAA GAG TGC AGA GTT ACT TAG ACC TTC CTC CGC AAT ACT CCC CCA GGT T-3′(Lapt), 3′-TAG ATG CTT AAG TAG TCC CGA TTT CTC ACG TCT CAA TGA ATC TGG AAG GAG GCG TTA TGA GGG GGT CCA A -5′(Aapt) were obtained from Sangon Biotechnology Inc. (Shanghai, China). Lysozyme was purchased from Sinopharm (China). ATP was purchased from Sangon Biotechnology Inc. (Shanghai, China). Other chemicals were all of analytical grade and used without any further purification. All solutions were prepared with Milli-Q deionized water.

### DNA Hybridization and Interaction with methyl violet (MV)

10.0 nmol of ATP aptamer and 10.0 nmol of lysozyme aptamer were added to 10 mM Tris (pH = 7.4), DNA hybridization was carried on for 40 min at 37 °C, then, 2.0 uM of MV was added to the solution to achieve a new complex Lapt≈Aapt-MV.

### Analyte Binding

Lysozyme and ATP at various concentration were added to the solution. The aptamer binding reaction was carried on for 20 min at 37 °C. A paralleled control experiment was always performed with no targets added.

### RLS Detection

The reacted solution was used to record the RLS spectrum which was obtained by synchronous scanning the excitation and emission monochromators (λex = λem) from 200.0 to 700.0 nm on a RF-5301PC fluorescence spectrophotometer. Both excitation and emission slit widths were kept at 3.0 nm.

## Conclusions

Herein, for the first time, we designed an aptasensor to one-spot simultaneously detect ATP and lysozyme. The preparation of the aptasensor was simple, including the obtaining of dsDNA and electronic interaction between MV and the dsDNA. During the procedure of detection, the corresponding side of the dsDNA will open when a target (lysozyme or ATP) “click” the aptamer. By the conbination of the RLS technique, it is found that under the optimal condition, the changed RLS intensity was proportional to the concentration of lysozyme and ATP. The detection limits of ATP and lysozyme were 10^−11^ M and 10^−12^ M. The aptasensor showed potential appliction for small molecule and protein detection by RLS, which could certainly extend the application of RLS technique. This sensor offers a rapid, selective and sensitive route for small molecule and protein detection and has good potential for wide use in practical applications.

### Supporting Information

Optimization of the assay conditions, including aptamer concentration, MV concentration, temperature, time and pH. The detailed descriptions of the bacterial culture and lysis.

## Additional Information

**How to cite this article**: Chen, F. *et al.* “Click on the bidirectional switch”: the aptasensor for simultaneous detection of lysozyme and ATP with high sensitivity and high selectivity. *Sci. Rep.* **6**, 18814; doi: 10.1038/srep18814 (2016).

## Supplementary Material

Supplementary Information

## Figures and Tables

**Table 1:** Detection of ATP and lysozyme in human serum.

ATP Content added	ATP content detected	Recovery (%)	Lysozyme content added	Lysozyme content detected	Recovery (%)
5 × 10^−9^	4.5350 × 10^−9^	90.7	1 × 10^−8^	9.968 × 10^−9^	99.68
1 × 10^−8^	9.380 × 10^−9^	93.8	5 × 10^−8^	5.125 × 10^− 8^	102.50
5 × 10^−8^	4.5995 × 10^−8^	91.99	1 × 10^−7^	1.081 × 10^−7^	108.10

**Table 2:** Simultaneous detection of ATP and lysozyme in bacterial lysates.

ATP Content added	Lysozyme content added	Δ*I*_RLS_ (323 nm)	Δ*I*_RLS_ (555 nm)	ATP Content detected	Lysozyme content detected
2 × 10^−8^	6 × 10^−8^	197.625	22.401	2.0296 × 10^−8^	6.3068 × 10^−8^
2 × 10^−8^	5 × 10^−9^	12.755	−34.283	2.0049 × 10^−8^	4.5239 × 10^−9^
5 × 10^−9^	5 × 10^−9^	31.17	−27.291	4.7031 × 10^−9^	5.6685 × 10^−9^
