# Supplementary material for: “Click on the bidirectional switch”: the aptasensor for simultaneous detection of lysozyme and ATP with high sensitivity and high selectivity
Source: Sci Rep. 2016 Jan 8;6:18814. doi: 10.1038/srep18814 (PMC4705532; doi:10.1038/srep18814)
Supplement: Supplementary Information [file srep18814-s1.doc]

**“****Click on the bidirectional switch”: the aptasensor for** **simultaneous detection of lysozyme and ATP with high sensitivity and high selectivity**

Feng Chen, Changqun Cai*, Xiaoming Chen, Chunyan Chen

Key Laboratory of Environmentally Friendly Chemistry and Applications of Ministry of Education, College of Chemistry, Xiangtan University, Xiangtan, Hunan 411105, China

Corresponding author. Tel.: +86 15273219560. Email addresses: cai_mao3@hotmail.com

Supporting Information includes: the effects of aptamer concentration, MV concentration, temperature, time and pH on the responses of the aptasensor.

Supplemental Figure 1: Study of the effect of aptamer concentration on the responses of the aptasensor. For lysozyme, ΔI_RLS_ (ΔI=I-I_0_); for ATP, ΔI_RLS_ (ΔI=I_0_-I).

Supplemental Figure 2: Study of the effect of MV concentration on the responses of the aptasensor

Supplemental Figure 3: Study of the effect of the time and temperature of DNA hybridization on the responses of the aptasensor

Supplemental Figure 4: Study of the effect of pH on the responses of the aptasensor. For lysozyme, ΔI_RLS_ (ΔI=I-I_0_); for ATP, ΔI_RLS_ (ΔI=I_0_-I).

Supplemental Figure 5: Study of the effect of the time of analyte binding on the responses of the aptasensor. For lysozyme, ΔI_RLS_ (ΔI=I-I_0_); for ATP, ΔI_RLS_ (ΔI=I_0_-I).

Bacterial culture and lysis

Escherichia coli lysate was used for the experiment described in the present work. After 36 hours incubation at 37 ^o^C, the bacteria were picked and suspended in 10mL of 0.9% NaCl. Subsequently, the suspension was centrifuged for 10 min (6000 rpm). Pellets were further sonicated. The resulting lysates were centrifuged for 10 min (14000 rpm) and supernatants were used for the experiment.
